# Pharmacokinetics and pharmacodynamics of intravenous artesunate during severe malaria treatment in Ugandan adults

**DOI:** 10.1186/1475-2875-11-132

**Published:** 2012-04-27

**Authors:** Pauline Byakika-Kibwika, Mohammed Lamorde, Jonathan Mayito, Lillian Nabukeera, Harriet Mayanja-Kizza, Elly Katabira, Warunee Hanpithakpong, Celestino Obua, Nadine Pakker, Niklas Lindegardh, Joel Tarning, Peter J de Vries, Concepta Merry

**Affiliations:** 1 Infectious Diseases Institute, Makerere University, P. O. Box 22418, Kampala, Uganda; 2 Department of Pharmacology and Therapeutics Trinity College, Dublin, Ireland; 3 Infectious Diseases Network for Treatment and Research in Africa, Kampala, Uganda; 4 Mahidol-Oxford Tropical Medicine Research Unit, Faculty of Tropical Medicine, Mahidol University, Bangkok, Thailand; 5 Department of Pharmacology and Therapeutics, Makerere University College of Health Sciences, Kampala, Uganda; 6 Centre for Clinical Vaccinology and Tropical Medicine, Churchill Hospital, Oxford, UK; 7 Division of Infectious Diseases, Tropical Medicine and AIDS, Academic Medical Center, Amsterdam, Netherlands

**Keywords:** Pharmacokinetics, Pharmacodynamics, Intravenous, Artesunate, Severe malaria

## Abstract

**Background:**

Severe malaria is a medical emergency with high mortality. Prompt achievement of therapeutic concentrations of highly effective anti-malarial drugs reduces the risk of death. The aim of this study was to assess the pharmacokinetics and pharmacodynamics of intravenous artesunate in Ugandan adults with severe malaria.

**Methods:**

Fourteen adults with severe falciparum malaria requiring parenteral therapy were treated with 2.4 mg/kg intravenous artesunate. Blood samples were collected after the initial dose and plasma concentrations of artesunate and dihydroartemisinin measured by solid-phase extraction and liquid chromatography-tandem mass spectrometry. The study was approved by the Makerere University Faculty of Medicine Research and Ethics Committee (Ref2010-015) and Uganda National Council of Science and Technology (HS605) and registered with ClinicalTrials.gov (NCT01122134).

**Results:**

All study participants achieved prompt resolution of symptoms and complete parasite clearance with median (range) parasite clearance time of 17 (8–24) hours. Median (range) maximal artesunate concentration (C_max_) was 3260 (1020–164000) ng/mL, terminal elimination half-life (T_1/2_) was 0.25 (0.1-1.8) hours and total artesunate exposure (AUC) was 727 (290–111256) ng·h/mL. Median (range) dihydroartemisinin C_max_ was 3140 (1670–9530) ng/mL, with T_max_ of 0.14 (0.6 – 6.07) hours and T_1/2_ of 1.31 (0.8–2.8) hours. Dihydroartemisinin AUC was 3492 (2183–6338) ng·h/mL. None of the participants reported adverse events.

**Conclusions:**

Plasma concentrations of artesunate and dihydroartemisinin were achieved rapidly with rapid and complete symptom resolution and parasite clearance with no adverse events.

## Background

Severe malaria is a medical emergency which if not treated results in 100% mortality. Mortality reduces to 15–20% with prompt, effective anti-malarial treatment and supportive care [1]. It is fundamental that plasma concentrations of a highly effective anti-malarial drug are achieved as rapidly as possible. Two classes of drugs are available for treatment of severe malaria; cinchona alkaloids, such as quinine, and artemisinin derivatives, such as artemether and artesunate.

Artesunate is a water-soluble hemisuccinate artemisinin derivative; available as sodium hemisuccinate salt for injection (Guilin Pharmaceutical Factory, Guangxi, People’s Republic of China). It has superior anti-malarial properties to quinine and artemether and studies have demonstrated a dramatic reduction in in-hospital mortality among children and adults treated with artesunate [2-7]. Very recent evidence strongly recommends intravenous artesunate as treatment of choice for severe falciparum malaria worldwide [1,2]. Artesunate’s excellent anti-malarial properties demonstrated by rapid parasite and fever clearance, is enhanced by its rapid hydrolysis to its active metabolite dihydroartemisinin [8-14].

Data on artesunate and dihydroartemisinin pharmacokinetic profiles have been reported in healthy volunteers [9,15,16] and patients with malaria [8,10,12,17-19] mainly in south-east Asia. However, data in African patients, who bear the brunt of malaria, are scarse. This study aimed to investigate the pharmacokinetics and pharmacodynamics of intravenous artesunate in Ugandan adults with severe malaria.

## Methods

### Study design and participants

Fourteen adults with severe malaria, admitted to Mulago National Referral Hospital, Kampala, Uganda were enrolled. Participants were enrolled consecutively if they were 18 years of age and above, with a positive blood smear for *Plasmodium falciparum* mono-infection, no other obvious cause of the fever or symptoms, with at least one laboratory or clinical feature of severe malaria and requiring parenteral therapy in accordance with the 2010 World Health Organization Guidelines [1]. Pregnant women, patients with history of anti-malarial intake within the last 72 hours and those receiving any herbal medication, known inhibitors or inducers of cytochrome P450 were excluded.

The study was approved by the Makerere University Faculty of Medicine Research and Ethics Committee (Ref2010-015) and Uganda National Council of Science and Technology (HS605) and registered with ClinicalTrials.gov (NCT01122134). Study procedures were explained to participants or their guardians in the local languages and information leaflets were provided. All participants provided written informed consent prior to enrollment.

### Study procedures

Participants were admitted to Mulago Hospital for treatment and monitoring. On admission, all participants received baseline evaluation including; thorough history, physical examination and laboratory investigations. All participants were weighed and blood samples were collected by finger-prick for malaria smears and venepuncture for haematocrit, plasma lactate, glucose, renal and liver function tests.

All participants received intravenous artesunate at a dose of 2.4 mg/kg at time 0, 2.4 mg/kg at 12 hours and 2.4 mg/kg/daily until they could tolerate oral therapy. Artesunate was dispensed in a 60-mg ampoule which was dissolved in 1 mL of 5% sodium bicarbonate to form sodium artesunate and diluted with 5 mL of 5% dextrose. The dose was injected as a slow bolus into an indwelling intravenous cannula over 3–4 minutes. Supportive therapy was given according to national malaria treatment guidelines. When participants could tolerate oral therapy, anti-malarial therapy was completed with a full three-day course of oral artemether-lumefantrine (Coartem®, Novartis Pharma AG, Basel, Switzerland). Participants administered oral therapy as unsupervised therapy at home but were given instructions to administer it with food or milk.

Serial thick blood films and measurement of parasite densities were performed until parasites were cleared following the schedule; 0, 0.5, 1, 2, 3, 4, 6, 8, 10, 12, 16, 18, 20, 24 hours and every six hours till six hours post-parasite clearance. Blood smears were stained with 2% Giemsa for thirty minutes and parasite densities calculated by counting the number of asexual parasites per 200 white blood cells (WBC) using the patient’s actual WBC count per uL of blood.

Blood for artesunate and dihydroartemisinin assays was drawn after the initial dose of artesunate in chilled fluoride-oxalate tubes from the arm opposite that used for drug administration at 0 (pre-dosing), 5, 10, 15, 30, 45 minutes, 1, 1.5, 2, 3, 4, 5, 6, 8 and 12 hours post-dosing. The twelfth hour sample was drawn pre-the twelve hour IV artesunate dose. Four mL of venous blood were collected at each sampling time. Blood samples were chilled immediately and transported to the laboratory on ice to avoid artesunate and dihydroartemisinin degradation. Samples were centrifuged within 30 minutes and the plasma was stored below −80°C until shipment on dry ice to the Clinical Pharmacology Laboratory at the Mahidol-Oxford Tropical Medicine Research Unit in Thailand for artesunate and dihydroartemisinin quantification. The maximum duration of sample storage was approximately three months at −80°C.

Complete physical examination was performed daily for each patient. Vital signs were monitored and recorded every four hours until parasite clearance. Participants were discharged home when they were afebrile, aparasitaemic and able to take oral therapy. They returned one week after discharge for review of symptoms, clinical examination and a repeat blood smear. Assessment of adverse events was performed on admission and 1 week post discharge using history and physical examination.

### Artesunate and dihydroartemisinin quantification

The plasma concentrations of artesunate and dihydroartemisinin were determined using solid-phase extraction and liquid chromatography-tandem mass spectrometry on an API 5000 triple-quadrupole mass spectrometer (Applied Biosystems/MDS SCIEX, Foster City, CA) with a TurboV ionization source operated in the positive ion mode [17]. Stable isotope-labelled artesunate and stable isotope-labelled dihydroartemisinin were used as internal standards. Total assay coefficients of variation for artesunate and dihydroartemisinin were <5% for inter- and intraday precisions. The lower limits of quantification (LLOQ) for artesunate and dihydroartemisinin were set at 1.2 and 2.0 ng/mL, respectively.

### Pharmacokinetic and statistical analysis

Pharmacokinetic analysis was performed with WinNonlin software, version 5.2 (Pharsight Corp., Mountain View, CA, USA) using an infusion non-compartmental analysis model. Complete bioconversion of artesunate to dihydroartemisinin was assumed. Calculated parameters included maximal observed concentration (C_max_), terminal elimination half-life (T_1/2_), total exposure measured as area under the plasma concentration-time curve (AUC_last_) from the start of drug infusion until the last quantifiable observation, elimination clearance (CL) and apparent volume of distribution (V). The AUC was calculated by application of the trapezoidal rule (linear-up/log-down). All parameters were calculated using time in hours after the time of first drug administration (T = 0). Drug concentrations below the LLOQ of the bioanalytical assays were treated as missing data.

Data were analysed using STATA® version 10.0 (StataCorp, College Station, TX). Baseline characteristics were summarized into medians with interquartile range (IQR). Pharmacokinetic parameters were summarized into medians with range. Parasite clearance time was defined as the time taken to clear all parasites from circulation ie time until the first of two sequential negative thick blood smears.

## Results

A total of 14 adults (9, 64% female) admitted with severe malaria were enrolled. At admission participants, had been ill for a median (IQR) of 7 (3 – 7) days. One participant had been ill for 14 and another 21 days. Some participants had more than one feature of severe malaria as follows two (14%) reported severe vomiting, five (36%) had jaundice, two (14%) had extreme weakness with inability to sit or stand, 10 (71%) were severely dehydrated, one (7%) had hyperpyrexia and one (7%) had haemoglobinuria. Median (range) parasite density at baseline was 18867 (500–79,950) parasites/μL. All participants received acetaminophen (paracetamol) for fever and pain relief. No other medications outside the study were administered. Baseline clinical and laboratory characteristics of study participants at admission are shown in Table 1.

**Table 1:** Baseline clinical and laboratory characteristics of study participants

**Parameter**	**Median (IQR)**
Age (years)	24.0 (20.0–35.0)
Weight (kgs)	56.3 (54–62.5)
Height (m)	1.7 (1.6–1.7)
BMI	20 (18.8–23.3)
Axillary temperature (^0^ C)	37.1 (36.5–38.8)
Respiratory rate ( per minute)	24.0 (24.0–26.0)
Pulse rate ( per minute)	106 (95–113)
Systolic blood pressure (mmHg)	114 (106–128)
Diastolic blood pressure (mmHg)	73 (69–82)
Hemoglobin (mg/dl)	12.9 (11.2–14.2)
Plasma creatinine (g/dl)	63.5 (54–75)
Serum bilirubin (μmol/L)	30 (15.2–87.6)
Serum lactate (mmol/L)	3.25 (1.9–4.1)
Blood sugar (mmol/L)	4.8 (4.4–6.1)

### Clinical response

**Figure 1:**
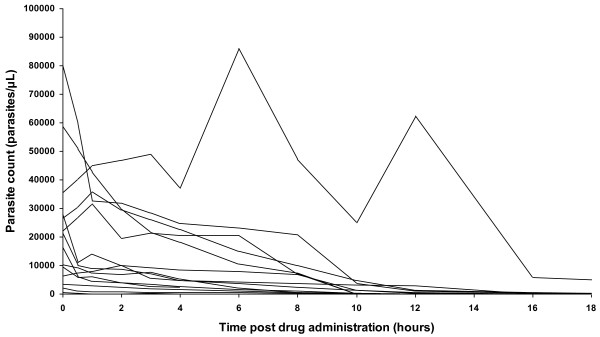
Individual plots of parasite density against time post start of treatment.

All study participants tolerated artesunate very well and reported very rapid recovery from symptoms and ability to take oral medication after 24 hours. No immediate adverse events were recorded. The median (range) parasite clearance time was 17 (8 – 24) hours. Individual plots of parasite density against time post start of treatment are shown in Figure 1. Blood smears for all participants remained negative for malaria parasites at one week post discharge from hospital. Only one patient had gametocytes at baseline and these cleared within 4 hours after start of treatment.

### Pharmacokinetics of artesunate and dihydroartemisinin

**Figure 2:**
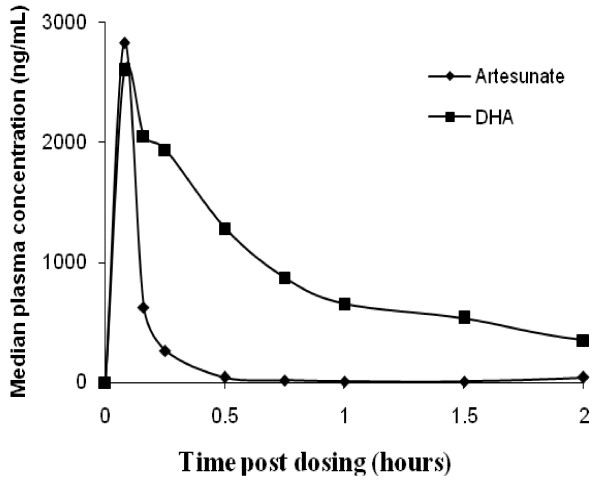
**Mean artesunate and dihydroartemisinin plasma concentration versus time.** Vertical bars represent standard error.

Pharmacokinetic parameters and profiles for artesunate and dihydroartemisinin are summarized in Table 2 and Figure 2. Following the intravenous administration, artesunate was detected in plasma very promptly rising to the C_max_ within a median (range) of 0.09 (0.6 – 6.07) hours.

**Table 2:** Pharmacokinetic parameters of artesunate and dihydroartemisinin

**Parameter**	**Artesunate**	**Dihydroartemisinin**
	**Median (range)**	**Median (range)**
Dose (mg)	140 (111–190)	103 (82–140)
C_max_ (ng/mL)	3260 (1020–164000)	3140 (1670–9530)
T_max_ (hr)	0.25 (0–6.07)	0.14 (0–6.07)
CL (L/hr)	180 (1–652)	32.25 (16–55)
V (L)	68.5 (0.18–818)	59.7 (26–117)
T_1/2_ (hr)	0.25 (0.11–1.82)	1.31 (0.89–2.87)
AUC_0-last_ (ng·h/mL)	727 (290–111256)	3492 (2183–6338)

It was cleared fast with median (range) elimination T_1/2_ of 0.25 (0.11 – 1.82) hours. Participants achieved the C_max_ for dihydroartemisinin within a median (range) of 0.14 (0.6 – 6.07) hours post dose administration (Table 2). There was no correlation between total artesunate or total dihydroartemisinin exposure with parasite clearance times (Spearman’s rho correlation coefficient −0.12 and −0.18 respectively).

## Discussion

This study aimed to investigate the pharmacokinetics and pharmacodynamics of intravenous artesunate in adults with severe *falciparum* malaria. Following intravenous administration of artesunate, study participants achieved plasma concentrations of artesunate and dihydroartemisinin very promptly. All participants achieved rapid parasite clearance with prompt resolution of symptoms and no adverse events.

High plasma concentrations of artesunate and dihydroartemisinin were achieved and artesunate was rapidly cleared from circulation. The C_max_ for artesunate and dihydroartemisinin were observed rapidly post-dose administration indicating rapid conversion of artesunate to dihydroartemisinin. Artesunate was cleared from circulation rapidly while dihydroartemisinin had a longer elimination T_1/2._ Previous studies have attributed the effectiveness of artesunate to its high initial C_max_ and rapid and extensive hydrolysis to dihydroartemisinin [8,14,18].

Although the peak concentration of artesunate was higher than that of dihydroartemisinin, total exposure to dihydroartemisinin was more than four times that of artesunate. The pharmacokinetic parameters observed in the present study are similar to findings from previous studies [8,10,11,14]. Both artesunate and dihydroartemisinin concentrations and AUC varied markedly among participants. This marked variability is similar to data from a previous study [19], however, despite the very large inter-individual variability all patients had very rapid parasite clearance. Parasite clearance time was comparable to that from a previous study [11] and shorter than a median of 66 hours from other studies [19,20]. The differences are possibly due to differences in parasite sensitivity or baseline parasitaemia.

Although a previous study suggested a trend of an association between artesunate and dihydroartemisinin AUC and parasite clearance [11]; the present study did not find this. The study by Newton *et al*, also demonstrated no relationship between artesunate pharmacokinetic parameters and parasiticidal effect [19]. It is not clear which artesunate pharmacokinetic parameter best correlates with anti-malarial treatment effect, but previous dose finding studies have suggested doses higher than 2 and 2.4 mg/kg as the minimum initial dose for malaria treatment in view of the considerable inter-individual variability in artesunate pharmacokinetic profile [19,20].

This study contributes to the existing knowledge on the clinical response to and pharmacokinetics of intravenous artesunate for treatment of severe malaria. Previous studies have demonstrated superiority of intravenous artesunate over intravenous quinine for severe malaria treatment [2,3]. These data strongly suggest that parenteral artesunate should be considered the drug of first choice in treating severe malaria [2,3]. Compared to artesunate, quinine has a number of disadvantages including poor compliance and a significant adverse event profile like hypotension, hypoglycaemia and gastro-intestinal intolerance [21,22]. The intravenous quinine infusion is difficult and expensive to institute and needs constant monitoring for arrhythmia and hypoglycaemia. Adherence to the eight-hourly regimen of intravenous quinine is poor especially in resource-limited settings and often patients do not complete the dose increasing the risks for treatment failure and development of drug resistance. The high patient-nurse ratio and lack of facilities for the IV infusion in health centers and hospitals in resource-limited settings lead to inappropriate and incorrect methods of quinine administration [23]. The ease of bolus intravenous administration plus the lack of a significant side effect profile make intravenous artesunate an excellent choice for use in very remote peripheral centers that suffer the greatest brunt of severe malaria. However, parenteral artesunate is not yet widely available and affordable especially in sub-Saharan Africa, where the greatest burden of severe malaria and death occurs and efforts to improve accessibility should be reinforced.

## Conclusions

Plasma concentrations of artesunate and dihydroartemisinin were achieved rapidly with rapid and complete symptom resolution and parasite clearance with no adverse events.

## Competing interests

The authors declare that they have no competing interests.

## Authors’ contributions

PBK, PJ D, CO and CM contributed to the design and conduct of the study. PBK, M L, JM and LN participated in recruitment of participants and data collection. WH and NL performed the bioanalytical assays. PBK, JT and NL analysed and interpreted the data. HMK, EK, NP, PJ D, and CM, participated in training the study staff and provided scientific support. PBK drafted the first version and all authors reviewed and approved the manuscript for submission.
